# Prevalence and Prognostic Impact of ASXL1 Somatic Mutation in Patients with Chronic Myeloid Leukemia: A Systematic Review and Meta-Analysis

**DOI:** 10.3390/cancers18132041

**Published:** 2026-06-24

**Authors:** Rita Ahmad, Motaz Almahmood, Rasha Kaddoura, Muhammad Ali Tariq, Ayman Abdullah Dalol, Marrita Rabadi, Aadhila Abbas Manthiri, Abdulrahman F. Al-Mashdali, Hatem Ahmed, Mohammed Abdulgayoom, Ayah Al Qaryoute, Sara Westall, Fadi Haddad, Shehab F. Mohamed

**Affiliations:** 1 Internal Medicine, University of North Dakota, Fargo, ND 58104, USA; 2 Tower Health, Internal Medicine, Phoenixville, PA 19460, USA; motaz.almahmood@towerhealth.org (M.A.); muhammadali.tariq@towerhealth.org (M.A.T.); hatem.sayedahmed@towerhealth.org (H.A.); 3 Pharmacy Department, Heart Hospital, Hamad Medical Corporation, Doha 3050, Qatar; rkaddoura@hamad.qa; 4 College of Medicine, Qatar University, Doha 2713, Qatar; ad2307825@qu.edu.qa (A.A.D.); mr2204570@qu.edu.qa (M.R.); 5 College of Pharmacy, Qatar University, Doha 2713, Qatar; aa2109872@qu.edu.qa; 6 Hematology Department, National Center for Cancer Care and Research (NCCCR), Doha 3050, Qatar; aalmashdali@hamad.qa (A.F.A.-M.); mmohammed35@hamad.qa (M.A.); smohamed22@hamad.qa (S.F.M.); 7 School of Medicine, Appalachian State University, Boone, NC 28608, USA; alqaryouteas@appstate.edu; 8 School of Medicine, University of North Dakota, Fargo, ND 58104, USA; sara.westall@und.edu; 9 MD Anderson Cancer Center, Department of Leukemia, Houston, TX 77030, USA; fhaddad@mdanderson.org

**Keywords:** chronic myeloid leukemia, CML, ASXL1 mutation, TKIs

## Abstract

**Background:** Outcomes in chronic myeloid leukemia (CML) remain heterogeneous despite effective BCR::ABL1 tyrosine kinase inhibitors (TKIs). Somatic mutations in epigenetic regulators, particularly additional sex combs-like 1 (ASXL1), have been implicated in adverse prognosis, but their clinical impact in CML has not been systematically defined. **Methods:** A systematic review was conducted using CINAHL, EMBASE, MEDLINE Ultimate, and PubMed from inception through August 2025. A total of 1339 records were identified; the eligible studies included adult and pediatric patients with chronic and advanced-phase (accelerated or blast) CML. After duplicate removal and screening, 11 studies met the inclusion criteria; these included adult patients only and were included in a qualitative synthesis and meta-analysis. ASXL1 mutation status was assessed using validated molecular methods. The outcomes included the molecular response, cytogenetic response, survival, and treatment resistance. Random-effects models were used to calculate the pooled odds ratios (ORs) with 95% confidence intervals (CIs). Statistical heterogeneity was assessed using the I^2^ statistic. **Results:** Across the included studies, ASXL1 mutations were detected in approximately 15% of patients. At 12 months, patients with ASXL1 mutations had significantly lower odds of achieving a major molecular response (MMR) compared with ASXL1-wildtype patients (OR 0.29; 95% CI 0.16–0.51; *p* < 0.0001; I^2^ = 30%). No statistically significant difference was observed in the complete cytogenetic response (CCyR) (OR 0.30; 95% CI 0.02–5.31; *p* = 0.41; I^2^ = 68%). Compared with patients harboring other non-ASXL1 somatic mutations, an ASXL1 mutation was not associated with a significant difference in MMR (OR 0.49; 95% CI 0.23–1.05; *p* = 0.067; I^2^ = 0%). **Conclusions:** ASXL1 mutations may be associated with an inferior molecular response to TKI therapy in CML, supporting their role as an adverse prognostic biomarker. These findings highlight the potential value of incorporating myeloid mutation profiling into future CML risk-stratification strategies.

## Simple Summary

Chronic myeloid leukemia (CML) is typically driven by a specific genetic change (BCR::ABL1) and is effectively treated with targeted therapies known as tyrosine kinase inhibitors (TKIs). However, some patients do not respond well to treatment or develop resistance over time. Recent advances in genetic testing have identified additional mutations, such as ASXL1, which may influence disease behavior and treatment outcomes. In this study, we systematically reviewed the available research to understand the clinical impact of ASXL1 mutations in patients with CML. Across 11 studies, we found that patients with ASXL1 mutations were less likely to achieve deep molecular responses, more likely to develop resistance to TKIs, and had higher rates of treatment failure compared with patients without these mutations. In several studies, ASXL1 mutations were also associated with faster disease progression and shorter survival. Overall, our findings suggest that ASXL1 mutations may represent a high-risk feature in CML. Identifying these mutations early could help guide closer monitoring and more personalized treatment strategies, with the goal of improving outcomes for patients.

## 1. Introduction

Chronic myeloid leukemia (CML) is a myeloproliferative neoplasm defined by the Philadelphia (Ph) chromosome and the BCR::ABL1 fusion gene, which encodes a constitutively active tyrosine kinase [1,2]. The introduction of tyrosine kinase inhibitors (TKIs), beginning with imatinib and subsequent second- and third-generation agents, has transformed CML into a largely chronic condition for many patients, with high rates of durable molecular responses and long-term survival [1,2,3,4]. Nevertheless, treatment outcomes remain heterogeneous: up to 10–15% of patients are resistant or intolerant to one or more TKIs, and progression to blast phase (BP) still occurs in approximately 5% of patients in the contemporary era [2,3,4].

Historically, the best-characterized mechanism of acquired TKI resistance is BCR::ABL1 kinase domain point mutations, which directly impair drug binding and guide the choice of subsequent TKIs [2,3,4]. However, such mutations account for only about half of resistance events, are rarely detectable at diagnosis in chronic phase (CP) CML, and therefore do not explain primary resistance [2,3,4]. A substantial fraction of resistance and disease progression appears to be BCR::ABL1-independent, driven by broader genomic instability, accumulation of additional cancer-related gene variants, and complex structural rearrangements. Integrative genomic analyses have shown that patients who later progress to BP or have poor outcomes often already harbor a higher burden of pathogenic or likely pathogenic variants in cancer-related genes at diagnosis than patients who achieve optimal molecular responses [5,6,7,8]. In addition, novel Ph-associated rearrangements, complex structural variants formed at the time of the Ph translocation and characterized by fragmentation, non-contiguous deletions, inversions and imperfect reassembly, have been described and linked to adverse outcomes and accelerated leukemic evolution [5,7,8].

Among non-ABL1 lesions, mutations in genes recurrently mutated in myeloid malignancies, particularly additional sex combs-like 1 (ASXL1), but also DNMT3A, TET2, RUNX1, TP53 and others, have emerged as important contributors to the genomic landscape of CML [4,9,10,11]. In BP-CML, the mutational profile appears to correlate with the blast phenotype: ASXL1, BCORL1, RUNX1 and TP53 mutations are more frequently associated with a myeloid blast phenotype, whereas CDKN2A/B and IKZF1 alterations are enriched in a lymphoid blast phase [4,9,10]. In the chronic phase, ASXL1 mutations have been reported in roughly 10–30% of patients across different cohorts, often co-occurring with other myeloid leukemia-associated mutations, but their clinical significance has only recently begun to be clarified [9,10,11,12,13]. Retrospective studies, discovery cohorts and more recent prospective or registry-based analyses have suggested that ASXL1 and other epigenetic regulator mutations are associated with slower or suboptimal molecular responses, higher rates of treatment failure, increased acquisition of BCR::ABL1 kinase domain mutations, and poorer survival on TKI therapy [4,11,12,13,14,15].

Despite accumulating genomic data, there are currently no universally adopted clinical guidelines for when and how to test for ASXL1 and other non-ABL1 mutations in CML; how to interpret such findings at diagnosis or in the setting of TKI resistance; and whether they should influence frontline TKI selection, monitoring intensity, or decisions regarding allogeneic transplantation. Recent expert recommendations and reviews have highlighted ASXL1 as a potential high-risk marker and suggested that its presence at diagnosis may not be fully mitigated by second-generation TKIs or asciminib-based strategies, although combination approaches are under investigation [2,14,15,16]. However, study designs, sequencing platforms, mutation thresholds, and treatment eras differ substantially between reports, making it challenging to draw firm, generalizable conclusions from individual studies and to translate them into routine clinical practice.

Given these uncertainties, there is a clear need for a systematic synthesis of the available evidence. The aim of this systematic review and meta-analysis is to study the prevalence and spectrum of ASXL1 mutations in chronic- and advanced-phase CML; evaluate the association between ASXL1 mutations and disease outcomes, including molecular response rates, progression to blast phase, and overall survival. By integrating data across studies and treatment eras, we seek to clarify the clinical impact of ASXL1 mutations in CML and to inform future diagnostic and therapeutic strategies, including the potential role of routine myeloid gene profiling at diagnosis and during the disease course.

## 2. Methods

### 2.1. Study Design

This systematic review and meta-analysis was conducted in accordance with the Preferred Reporting Items for Systematic Reviews and Meta-Analyses (PRISMA) guidelines [17]. The objective was to evaluate the prevalence and prognostic impact of ASXL1 somatic mutations in patients with chronic myeloid leukemia (CML) across disease phases and treatment eras. The study was registered in the INPLASY system with number INPLASY202630075.

### 2.2. Search Strategy

A comprehensive literature search was performed by a medical librarian across CINAHL, EMBASE, MEDLINE Ultimate, and PubMed from database inception through August 2025. The detailed search strategies for each database are provided in the Supplementary Table S1. The search was restricted to English-language publications. The reference lists of eligible studies were manually screened to identify additional relevant reports.

### 2.3. Eligibility Criteria

Studies were eligible if they met the following criteria:

1. Included adult patients diagnosed with Philadelphia chromosome-positive CML according to World Health Organization or European LeukemiaNet criteria.
2. The reported ASXL1 mutation status was assessed using validated molecular techniques, including next-generation sequencing or polymerase chain reaction-based assays.
3. Evaluated either the prevalence of ASXL1 mutations or their association with clinical outcomes, including molecular response, cytogenetic response, progression, treatment resistance, or survival.
4. Included patients in chronic, accelerated, or blast-phase CML.

We excluded review articles, editorials, commentaries, guidelines, case reports, small case series, and preclinical or basic science studies. Studies that included diseases other than CML without separate CML-specific outcome reporting were also excluded.

### 2.4. Study Selection

All records were imported into a citation management system and duplicates were removed. Titles and abstracts were independently screened by two reviewers (RA and MA). Full-text review was subsequently performed for potentially eligible studies. Discrepancies were resolved by consensus.

### 2.5. Data Extraction

Data extraction was performed independently by five investigators (RA, MA, AD, MR and AAM) using a standardized, predesigned extraction form. Extracted variables included study characteristics (design, country, sample size), patient demographics, disease-phase distribution, ASXL1 detection method, treatment exposure, and reported outcomes. Clinical endpoints included major molecular response (MMR), complete cytogenetic response (CCyR), progression, resistance, and survival outcomes where available. Disagreements were resolved through discussion with senior reviewers.

### 2.6. Risk of Bias Assessment

Risk of bias/quality assessment was performed independently by two reviewers using the Newcastle–Ottawa Scale (NOS) (modified as applicable for observational studies). NOS evaluates studies across three domains: selection (maximum 4 stars), comparability (maximum 2 stars), and outcome (maximum 3 stars), with maximum total score of 9 stars [18]. Any discrepancies between two reviewers were resolved through discussion and consensus (with senior reviewer adjudication if needed). Based on total NOS score, studies were categorized as good quality (7–9 stars), fair quality (4–6 stars), or poor quality (0–3 stars). Detailed study-level ratings are presented in Table 1 below.

### 2.7. Statistical Analysis

This is a study-level meta-analysis. The odds ratios (ORs), with their respective 95% confidence intervals (95% CIs), were computed. The minimum number considered for performing a quantitative analysis was two. A random-effects model was chosen to account for the anticipated heterogeneity between the studies. The inconsistency factor (*I*^2^) was used to assess the heterogeneity between studies with values greater than 50% to represent high heterogeneity. Funnel plots to visually inspect the publication bias were not included and Egger’s test was not conducted because the number of the studies from which the clinical outcomes were pooled was less than 10. A sensitivity analysis was performed to investigate the impact of CP-CML on the total effect size and heterogeneity when applicable, using a leave-one-out approach. A single-proportion meta-analysis was performed to pool the prevalence data. A funnel plot was included for the prevalence variable. A *p*-value of <0.05 was set as statistical significance. The statistical program R software (RStudio 2023.06.0+421 “Mountain Hydrangea” Release for windows) was utilized to conduct the analysis.

## 3. Results

### 3.1. Study Selection

**Figure 1:**
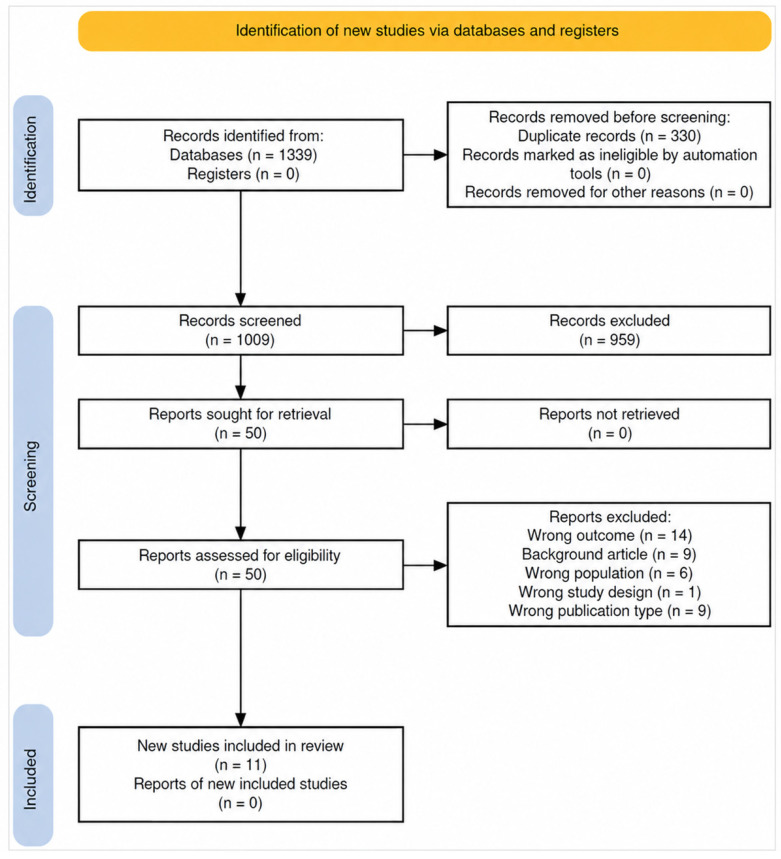
Flow diagram of study selection. PRISMA checklist see File S1.

Following the PRISMA guidelines, 1339 records were identified through database searching. After the removal of 330 duplicates, 1009 records were screened by title and abstract, and 50 full-text articles were assessed for eligibility. Eleven observational studies met the inclusion criteria and were included in the qualitative synthesis and quantitative meta-analysis [9,19,20,21,22,23,24,25,26,27,28] (Figure 1). One potential study was excluded [14] because of an overlapping patient population with the population of an included study [27]. Only five of the included studies had comparison groups that investigated the impact of ASXL1 mutation on the outcomes [19,20,21,22,27]. Three of them reported the outcomes of CP-CML patients [19,21,27]. The remaining two studies reported the outcomes for patients in all phases [20,22].

### 3.2. Study Characteristics

The included studies encompassed adult patients (38–64 years old) with CML diagnosis according to the World Health Organization (WHO) [29] or European LeukemiaNet (ELN) criteria [2], and included patients across chronic, accelerated, and blast phases with CP predominance. Their ASXL1 mutation status was assessed using next-generation sequencing, polymerase chain reaction (PCR)-based assays, or other validated molecular techniques. Most studies evaluated the outcomes in patients treated with first- or later-generation TKIs, with variability in the treatment era, sequencing depth, and timing of mutation assessment. The included cohorts varied in size (22–515 patients), ranging from small single-center studies to larger multicenter or registry-based analyses, and included both newly diagnosed and previously treated patients (Table S2).

### 3.3. Prevalence of ASXL1 Mutations

**Figure 2:**
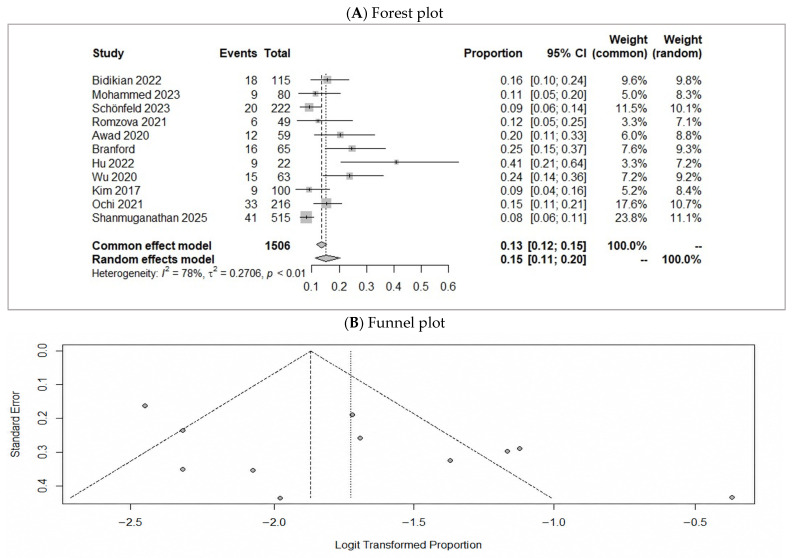
Prevalence of ASXL1 mutations [9,10,19,20,21,22,23,24,25,26,28].

Across the 11 studies contributing to prevalence pooling (n = 1506) [9,19,20,21,22,23,24,25,26,27,28], the pooled prevalence of ASXL1 mutations across all CML phases was 15% (95% CI, 11–20%), with substantial heterogeneity between studies (*I*^2^ = 78%, *p* < 0.01) (Figure 2, Panel A). The funnel plot shows some asymmetry indicating potential publication bias (Figure 2, Panel B). The sensitivity analysis, which removed the two studies that only recruited patients with CP-CML [21,27], yielded a prevalence of 18%, a 95% CI of [13–23%], and *I*^2^ = 61% (Figure S1). The prevalence of ASXL1 mutation in patients with CP-CML was 12%, with a 95% CI of 7–18%, and *I*^2^ = 76% (Figure S2). The individual studies’ prevalence estimates ranged from approximately 8% to 41%, reflecting the heterogeneity in patient populations, disease-phase distribution, age, and sequencing methodologies. Despite this variability, ASXL1 mutations were consistently identified across all included cohorts.

### 3.4. Impact of ASXL1 Mutations on Treatment Response

**Figure 3:**
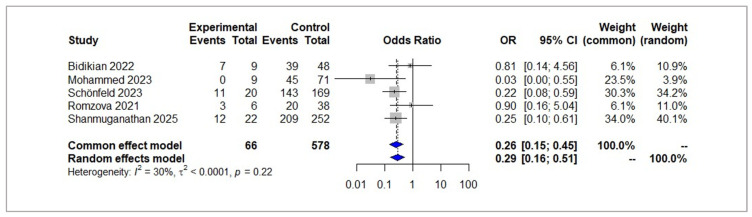
Forest plot for major molecular response at 12 months (ASXL1 mutation versus no mutation) [10,19,20,21,22].

**Figure 4:**
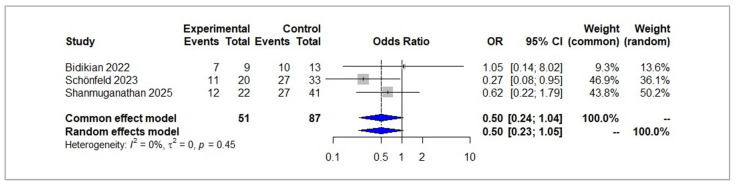
Forest plot for major molecular response at 12 months (ASXL1 mutation versus other mutations) [10,19,21].

**Figure 5:**
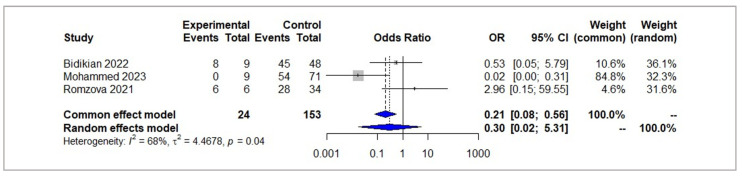
Forest plot for complete cytogenetic response (ASXL1 mutation versus no mutation) [19,20,22].

Among the reported clinical outcomes, only the major molecular response (MMR) and complete cytogenetic response (CCyR) were mutually reported and could be pooled. The ASXL1 mutation status was associated with an inferior molecular response to TKI therapy. In the pooled analysis comparing patients with an ASXL1 mutation versus an ASXL1 wildtype (i.e., no mutation), the odds of achieving MMR at 12 months were significantly lower among the patients with ASXL1 mutations (OR 0.29; 95% CI 0.16–0.51; *p* < 0.0001), corresponding to a 71% reduction in the likelihood of achieving MMR. The between-study heterogeneity for this outcome was low-to-moderate (*I*^2^ = 30%), indicating consistent findings across studies (Figure 3). The sensitivity analysis conducted by including the studies that reported the outcomes in patients with CP-CML [19,21,27] yielded comparable results (OR 0.28; 95% CI: 0.15–0.51; *p* < 0.0001; *I*^2^ = 0%) without heterogeneity (Figure S3). However, the sensitivity analysis that included studies reporting the outcomes of CML patients across all phases [20,22] did not show statistical difference between the comparison groups (OR 0.20; 95% CI: [0.01–5.46]; *p* = 0.3434; *I*^2^ = 74%) (Figure S4). When patients with ASXL1 mutations were compared with patients harboring other non-ASXL1 somatic mutations, there was no statistically significant difference in the 12-month MMR rates (OR 0.49; 95% CI 0.23–1.05; *p* = 0.067), with no observed heterogeneity (*I*^2^ = 0%) (Figure 4). In contrast, the CCyR did not differ significantly by ASXL1 mutation status (OR 0.30; 95% CI 0.02–5.31; *p* = 0.41), although the heterogeneity was substantial (*I*^2^ = 68%) (Figure 5), suggesting variability in study design, patient characteristics, and CCyR assessment. The sensitivity analysis performed by removing the study by Bidikian et al. [19], which reported the outcomes in patients with CP-CML, yielded similar non-significant results (OR 0.22; 95% CI: [0.0014–34.8579]; *p* = 0.5583; *I*^2^=83%) (Figure S5).

### 3.5. Additional Non-Pooled Outcomes: Treatment Resistance, Failure, Progression, and Survival

Only the MMR and CCyR were sufficiently and consistently reported for a meta-analysis. Other clinically important outcomes—TKI resistance/kinase-domain mutations, treatment failure composites, progression, and survival endpoints—were reported inconsistently across studies and are therefore summarized narratively.

For resistance/kinase-domain mutations, Shanmuganathan et al. [27] reported a higher frequency of TKI-resistant BCR::ABL1 kinase-domain mutations at 2 years in patients with ASXL1 mutations (35%) compared with patients with other mutations (2%) or no mutations (1%) (*p* < 0.001). The same study reported lower 2-year event-free survival (EFS) in patients with ASXL1 mutations versus no mutations (61% vs. 91%, *p* < 0.001). Rafiq Mohammed et al. [20] reported imatinib resistance in 9/9 (100%) patients with ASXL1 mutations compared with 26/71 (36.6%) ASXL1-wildtype patients (*p* = 0.01), and nilotinib resistance in 4/9 (44.4%) patients with ASXL1 mutations.

Treatment failure endpoints: Kim et al. [24] reported that a composite endpoint of treatment failure or failure to achieve/maintain CCyR at 12 months occurred in 5/9 (55.6%) patients with ASXL1 mutations versus 7/63 (11.1%) patients without mutations (*p* = 0.015). Branford et al. [9] reported a poor outcome/TKI failure in 7/9 (78%) patients with ASXL1 mutations in an imatinib-era cohort.

Progression outcomes were mainly reported by Ochi et al. [25], with a significantly shorter time to blast phase progression among patients with ASXL1 mutations (HR 4.66; 95% CI 1.99–10.89; *p* < 0.001). Additionally, Branford et al. [9] reported progression to blast crisis in 6/9 patients with ASXL1 mutations.

Moreover, survival and time-to-event outcomes were provided by Bidikian et al. [19], who reported phase-dependent outcomes in patients with ASXL1 mutations. There was an overall survival (OS) of 7.2 months in BP-ASXL1 and 25.7 months in AP-ASXL1; the 5-year progression-free survival (PFS) was 88% in CP-ASXL1 compared with 0% in BP-ASXL1 and 24% in AP-ASXL1. Romzova et al. [22] reported no statistically significant association between ASXL1 status at diagnosis and a poorer molecular response or higher treatment failure in their cohort (Table 2). demonstrates additional non-pooled outcomes that were reported in the studies included in this review.

## 4. Discussion

CML represents a paradigm of successful targeted therapy; however, clinically meaningful heterogeneity persists in treatment responses despite effective BCR::ABL1 inhibition. Early molecular response milestones, particularly achievement of major molecular response (MMR), remain central to contemporary management, as they strongly predict long-term outcomes, including progression-free survival, overall survival, and the likelihood of achieving deep molecular response (DMR) and treatment-free remission (TFR). Within this framework, identifying the biologic factors that influence treatment response kinetics is of high clinical relevance [1,2,3].

This is supported by landmark outcome studies demonstrating that their levels at 3, 6, and 12 months predict the probability of achieving CCyR and MMR, and are strongly associated with long-term survival endpoints [4]. Beyond survival, the early molecular response functions as a “gateway” to DMR, an essential step for TFR, because patients who do not reach timely MMR are substantially less likely to achieve and sustain MR4/MR4.5, which are used in most discontinuation frameworks [1,2,3,5,6,7]. Within this framework, identifying the biologic factors that influence treatment response kinetics is of high clinical relevance [6,7].

In this systematic review and meta-analysis, we evaluated the association between ASXL1 mutations and treatment response to tyrosine kinase inhibitors (TKIs), as well as their broader clinical implications. Our pooled analysis demonstrates that ASXL1 mutations are associated with significantly reduced odds of achieving MMR at 12 months. This finding supports a predictive role of ASXL1 mutations in identifying patients less likely to achieve an optimal molecular response with TKI therapy. In parallel, several included studies reported associations between ASXL1 mutations and adverse clinical outcomes, including disease progression, treatment resistance, and inferior survival, supporting an additional prognostic role [8,9,10,11,12].

ASXL1 mutations represent a modern molecular challenge of the poor-prognosis clonal evolution long recognized in CML. Cytogenetic clonal evolution (CE) was established as an independent poor prognostic factor in imatinib-treated patients, with inferior 2-year survival (77% vs. 92%, *p* = 0.002) even when CE occurred as the sole accelerated-phase criterion [30]. ASXL1 mutations function as a molecular equivalent of this CE phenomenon detectable by next-generation sequencing, occurring in 7–14% of chronic-phase patients at diagnosis [14,19,21,27], and rising dramatically to approximately 40% in the accelerated phase [19]. Unlike other clonal hematopoiesis-associated variants that remain relatively indolent, ASXL1 hotspot mutations, predominantly frameshift and nonsense variants clustered around codon G646, are disproportionately enriched in myeloid diseases compared with clonal hematopoiesis of indeterminate potential (CHIP), suggesting that they are inherently pathogenic and confer a high risk of disease progression [31].

The mechanism by which ASXL1 mutations drive this BCR::ABL1-independent resistance involves dual pathways. ASXL1 truncation mutations alter the polycomb repressive deubiquitinase (PR-DUB) complex’s function through gain-of-function effects on BAP1 deubiquitinase activity [31,32], leading to aberrant histone H2A ubiquitination, phosphorylated AKT stabilization, and impaired DNA damage response [32]. In parallel, recent evidence demonstrates that CML cells with mutant ASXL1 upregulate the ALOX5–BLT receptor signaling pathway, creating a BCR::ABL1-independent survival mechanism that sustains leukemic cells despite effective TKI-mediated BCR::ABL1 inhibition [33].

In broader hematology, ASXL1 is classically associated with adverse phenotypes in several myeloid disorders, consistent with its role in epigenetic regulation and stem/progenitor cell programs [14,15]. However, the interpretation of ASXL1 variants requires additional nuance because ASXL1 is also a canonical driver of age-related CHIP. Large population studies have demonstrated that DNMT3A, TET2, and ASXL1 mutations accumulate with age in otherwise healthy individuals, with CHIP prevalence rising substantially in older populations and being linked to nonmalignant risks, such as cardiovascular disease [24,34,35].

In CML specifically, ASXL1 has repeatedly emerged as one of the most frequent non-BCR::ABL1 lesions detected at diagnosis, and is associated with inferior response kinetics and adverse failure-related endpoints. In an integrative genomic analysis of newly diagnosed patients, cancer-associated mutations at diagnosis including ASXL1 were enriched among patients with poor outcomes [9]. Additional diagnostic studies have confirmed that mutated cancer-related genes at diagnosis have measurable clinical impacts, reinforcing the notion that early genomics may capture the underlying evolutionary risk [10,11]. In a large clinical cohort focused on CP-CML, Bidikian and colleagues reported ASXL1 as the most frequent mutation and demonstrated significantly worse event-free and failure-free survival in ASXL1-mutated CP-CML, with ASXL1 remaining an independent adverse predictor in a multivariable analysis [19]. Prospective trial-adjacent evidence also supports a response kinetics signal: in the prospective German TIGER study (CML-V; NCT01657604), in the cohort treated with nilotinib-based therapy, ASXL1 was the most frequent mutation and predicted an inferior molecular response across multiple time points [21].

We found that ASXL1 mutations were not rare, but their prevalence estimates were heterogeneous across studies, consistent with the differences in age structure, disease phase inclusion, testing indications (diagnosis vs. resistance evaluation), and sequencing platforms/thresholds. Importantly, our pooled analysis demonstrates a strong association between ASXL1 and reduced likelihood of achieving MMR at 12 months, an endpoint with high clinical and translational relevance because it is tightly linked to downstream DMR attainment, long-term failure risk, and the feasibility of TFR [1,2,3,4,5,6,7]. In contemporary management, failure to reach MR3/MMR by 12 months is not simply a “late responder” phenotype; it often signals the need for intensified evaluation (adherence, drug interactions, mutation testing) and potentially early therapeutic optimization, and it materially reduces the probability of later achieving the sustained DMR required for safe discontinuation attempts [1,2,3,5,6,7].

Furthermore, ASXL1 mutations were associated with adverse outcomes beyond molecular response. Across several cohorts, ASXL1 was enriched in TKI resistance and BCR::ABL1 kinase-domain mutations, and studies using composite clinical endpoints reported higher rates of treatment failure in patients with ASXL1 mutations. Multiple datasets also linked ASXL1 to disease evolution, including a shorter time to blast phase progression, and phase-stratified analyses showed markedly inferior survival once ASXL1-mutated disease entered the accelerated/blast phase. However, not all cohorts reproduced these associations, underscoring the heterogeneity in patient populations, sequencing indications, TKI exposure, and endpoint definitions [9,25]. Collectively, these findings support the potential integration of ASXL1 status into risk stratification and highlight the need for standardized outcome reporting in future studies.

One of the most clinically intriguing developments in the modern literature, and an important lens for interpreting our findings, is the evidence that ASXL1 variants at diagnosis may enrich for future resistance evolution, particularly BCR::ABL1 kinase domain (KD) mutation acquisition. In a frontline “potent TKI” study using nilotinib, dasatinib, or asciminib, ASXL1 variants at diagnosis were associated with significantly lower 12-month MMR and worse failure-free survival, and were strikingly enriched for subsequent KD mutations, suggesting an evolution-prone state that is not fully mitigated by upfront potent BCR::ABL1 inhibition [27].

An apparent discrepancy in outcome interpretations is reflected in the pooled CCyR endpoints, which appear less consistent than the molecular endpoints. The pooled estimate for CCyR was associated with a very wide confidence interval (0.02–5.31) and substantial heterogeneity, indicating a high degree of statistical uncertainty. Therefore, no definitive conclusion can be drawn regarding the relationship between ASXL1 mutations and CCyR based on the currently available data. This discrepancy between molecular and cytogenetic endpoints likely reflects differences in sensitivity, timing of assessment, and evolving clinical practice, where molecular monitoring has largely supplanted cytogenetics [1,2,3]. Notably, early molecular milestones (including 3-month EMR and 12-month MMR) have repeatedly shown durable prognostic value for survival endpoints and for deeper molecular responses, supporting the primacy of molecular outcomes when evaluating genomic risk factors such as ASXL1 [1,2,3,4,21,27].

Several biologically plausible hypotheses can be advanced to explain why ASXL1 is associated with inferior early MMR and higher failure-related risk. First, ASXL1 may tag a stem cell-biased disease state with increased persistence of leukemic progenitors under TKI pressure, leading to slower clearance of BCR::ABL1 transcripts and delayed achievement of molecular milestones [9,10,11,13,14,15]. Second, ASXL1 may reflect a higher-evolutionary-capacity state, either through broader genomic complexity or altered chromatin programs that facilitate selection of resistant clones, consistent with integrative genomic studies linking additional mutations at diagnosis to poor outcomes, and with modern data linking ASXL1 to KD mutation acquisition [9,10,11,12,27]. Third, CHIP overlap likely contributes to heterogeneity: in older adults, a low-VAF ASXL1 clone may be Ph-negative and persist even when BCR::ABL1 is deeply suppressed, complicating the binary “mutation-positive” interpretation and raising questions about long-term implications for survivorship and TFR. This concern is supported by studies describing persistent or dynamic non-BCR::ABL1 somatic variants during TKI therapy and deep molecular response, underscoring that the clonal context matters and that not all ASXL1 detections are equivalent [24].

These findings have several implications regarding generalizability and future research. First, our data support ASXL1 as a clinically meaningful biomarker for impaired early molecular response, which is itself a cornerstone predictor of long-term outcomes and a practical prerequisite for DMR and TFR attempts [1,2,3,4,5,6,7]. Second, standardization is needed: sequencing panels, VAF thresholds, timing of testing, and endpoint definitions vary widely and likely drive prevalence heterogeneity, emphasizing the importance of harmonized reporting and careful subgroup analyses by phase, age, and testing indication [1,2,3,9,10,11,12]. Third, prospective studies should evaluate whether integrating myeloid mutation profiling at diagnosis improves risk stratification beyond clinical scores, and whether ASXL1-positive patients benefit from intensified molecular monitoring, earlier KD testing, earlier switching strategies, or rational combination approaches (e.g., asciminib plus ATP-competitive TKIs) in genomically high-risk subgroups [1,2,3,27,36,37]. Finally, clonal tracking studies are essential to disentangle disease-related ASXL1 from CHIP-related ASXL1, to map how variant allele fractions evolve under TKI therapy, and to determine whether persistent Ph-negative ASXL1 clones influence the safety and durability of TFR in patients who otherwise meet the molecular eligibility criteria [6,7,24]. In summary, our meta-analysis shows potential evidence that ASXL1 may be a clinically relevant risk marker in CML, primarily through its association with inferior early MMR [1,2,3,4,5,6,7].

This meta-analysis has several methodological limitations that should be acknowledged. As a study-level synthesis, it is inherently more susceptible to inter-study heterogeneity than an individual patient data meta-analysis. Only 11 studies were identified, predominantly observational in design with small sample sizes, rendering the findings prone to confounding and bias and potentially compromising their validity. A random-effects model was therefore employed to account for between-study variability. Moreover, the number of studies included in the quantitative analyses was limited, and some outcomes were derived from a small number of cohorts, which may have affected the robustness of the pooled estimates. The inclusion of heterogeneous disease phases and treatment regimens may have introduced potential confounding. In addition, the restriction to English-language studies may have introduced selection bias. The sensitivity analysis shows that the heterogeneity regarding the CML phase may have affected the results. Performing a meta-regression for the comparative analysis was not feasible due to the small number of studies included (i.e., <10 studies). Finally, the inability to perform subgroup analyses based on the TKI type limited a more granular interpretation. Consequently, the findings of this meta-analysis should be interpreted with caution.

## 5. Conclusions

Despite our study’s limitations, our findings demonstrate that ASXL1 mutations may be associated with an inferior molecular response to TKI therapy in CML. These results support further investigation into the integration of myeloid mutation profiling into CML risk stratification and highlight the need for prospective, standardized studies to better define the clinical utility of ASXL1 in guiding treatment decisions.

## Figures and Tables

**Table 1:** Study-level ratings.

Study (Year)	Selection (Max 4★)	Comparability (Max 2★)	Outcome (Max 3★)	Total Score	Quality Level
**Adnan Awad et al. (2020) [8]**	★★★★	★★	★★★	9	Good
**Branford et al. (2018) [9]**	★★★★	★★	★★★	9	Good
**Bidikian et al. (2022) [19]**	★★★★	★★	★★★	9	Good
**Rafiq Mohammed et al. (2023) [20]**	★★★	★	★★	6	Fair
**Schönfeld et al. (2022) [21]**	★★★★	★★	★★★	9	Good
**Romzova et al. (2021) [22]**	★★★	★	★★	6	Fair
**Hu et al. (2022) [23]**	★★★	★	★★	6	Fair
**Kim et al. (2017) [24]**	★★★	★	★★	6	Fair
**Ochi et al. (2021) [25]**	★★★★	★★	★★★	9	Good
**Wu et al. (2020) [26]**	★★★	★	★★	6	Fair
**Shanmuganathan et al. (2025) [27]**	★★★★	★★	★★★	9	Good

**Table 2:** Non-pooled outcomes reported in included studies (resistance, failure, progression, and survival).

Study	Country/Design	N (Phase)	ASXL1 n (%)	TKI Context	Molecular Response	TKI Resistance	Progression	Survival
Shanmuganathan 2025 [10]	Australia, NZ Retrospective	515 (CP)	40 (8%)	Mixed TKIs	MMR at 12 m: 55% vs. 83%, *p* = 0.033	TKI-resistant mutations at 2 year: 35% vs. 1%, *p* < 0.001	-	2 year EFS: 61% vs. 91%, *p* < 0.001
Mohammed 2023 [20]	Iraq, Iran Retrospective	80 (CP/AP/BP)	9 (11.3%)	Mixed TKIs	MMR at 12–24 m: 0%	Imatinib: 100% vs. 36.6%, *p* = 0.01; Nilotinib: 44.4%	-	-
Bidikian 2022 [19]	USA Multicenter retrospective	115 (CP/AP/BP)	21 (18.3%)	1st- and later- gen TKIs	CCyR: CP, 89%; AP, 33%; BP, 20%; MMR CP: 78%, median 17.5 m	45–50% failed MMR	-	OS BP: 7.2 m; OS AP: 25.7 m; 5 year PFS: CP, 88%; AP, 24%; BP, 0%
Schönfeld 2022 [21]	Germany Prospective	222 (CP)	20 (9%)	Frontline imatinib	MMR at 12 m: 55% vs. 85%; 18 m: 60% vs. 89%; 24 m: 65% vs. 89%, *p* < 0.008	-	-	-
Hu 2022 [23]	China Retrospective	22 (CP)	9 (40.9%)	Mixed TKIs	No MMR difference at 12 m; MR4.0 inferior at 36 m	-	-	-
Romzova 2021 [22]	Czech Republic Prospective	49 (CP)	6 (12.2%)	Mixed TKIs	No significant molecular response difference	-	-	-
Ochi 2021 [25]	Japan Multicenter cohort	216 (CP/BC)	33 (15.3%)	Mixed TKIs	-	-	Time to blast phase HR: 4.66 (95% CI 1.99–10.89), *p* < 0.001	-
Awad 2020 [28]	Finland, Egypt Retrospective	59 (CP/AP)	11 (18.6%)	Mixed TKIs	Poor outcomes in co-mutated cases; exact rates not reported	-	-	-
Wu 2020 [26]	China Cross-sectional genomic cohort	63 (CP/AP resistant)	15 (23.8%)	Resistant or intolerant	No CCyR/MMR data	Resistance-enriched cohort	No independent PFS impact	No independent PFS impact
Branford 2018 [9]	Australia, Germany, UK, and Korea Retrospective	65 (CP/BC)	9 (13.8%)	Frontline imatinib	MMR3 2/9	7/9 TKI failure	6/9 progressed to BC	-
Kim 2017 [24]	South Korea Retrospective	100 (CP/AP/BP)	9 (9%)	Imatinib based	12 m CCyR failure: 55.6% vs. 11.1%, *p* = 0.015; 24 m MMR: 44.4% vs. 88.9%	Treatment failure: 55.6%	-	-

Abbreviations: CP, chronic phase; AP, accelerated phase; BC, blast crisis; BP, blast phase; CCyR, complete cytogenetic response; EFS, event-free survival; MMR, major molecular response; OS, overall survival; PFS, progression-free survival; TKI, tyrosine kinase inhibitor.

## Data Availability

The original contributions presented in this study are included in the article/Supplementary Material. Further inquiries can be directed to the corresponding author.

## Supplementary Materials

The following supporting information can be downloaded at: https://www.mdpi.com/article/10.3390/cancers18132041/s1, File S1: PRISMA checklist; Table S1: Search strategy; Table S2: Study characteristics; Figure S1: Forest and funnel plots for the prevalence of ASXL1 mutations. Sensitivity analysis by removing studies by Schönfeld 2023 and Shanmuganathan 2025 (i.e., patients with CP-CML); Figure S2: Forest and funnel plots for the prevalence of ASXL1 mutations in CP-CML; Figure S3: MMR—Sensitivity analysis using studies by Bidikan, Schonfeldl, and Shanmuganathan et al. (i.e., patients with CP-CML only); Figure S4: MMR—Sensitivity analysis by removing studies by Bidikan, Schonfeld, and Shanmuganathan et al. (i.e., patients with CP-CML); Figure S5: CcyR—Sensitivity analysis by removing study by Bidikan et al. (i.e., patients with CP-CML).
