# Supplementary material for: Prevalence and Prognostic Impact of ASXL1 Somatic Mutation in Patients with Chronic Myeloid Leukemia: A Systematic Review and Meta-Analysis
Source: Cancers (Basel). 2026 Jun 24;18(13):2041. doi: 10.3390/cancers18132041 (PMC13360061; doi:10.3390/cancers18132041)
Supplement: Supplementary file 1 [file cancers-18-02041-s001.zip › cancers-4393193-supplementary Tables & Figures.pdf]

Supplementary Material

Tables

Table S1. Search strategy.

Database	Search Strategy
CINAHL	Filtered to English Language: (XB Additional Sex Combs Like 1 OR XB asxl1 OR XB asxl1 mutat* OR XB asxl1 alterat* OR XB asxl1 delet* OR XB asxl1 loss) AND (XB MH "Leukemia, Myeloid, Chronic" OR XB Myeloproliferative Neoplasms OR XB MPN OR XB Myeloproliferative Disorder OR XB CML OR XB BCR-ABL1 positive MPN) AND (XB MH "Mutation" OR XB mutat* OR XB genetic alterat* OR XB somatic mutat* OR XB variant OR XB variants OR XB delet* OR XB loss of heterozygosity OR XB LOH)
EMBASE	('additional sex combs like 1':ti,ab,kw OR 'asxl 1':ti,ab,kw OR 'asxl1 mutat*':ti,ab,kw OR 'asxl1 alterat*':ti,ab,kw OR 'asxl1 delet*':ti,ab,kw OR 'asxl1 loss':ti,ab,kw) AND ('chronic myeloid leukemia'/exp OR 'chronic myeloid leukemia'/de OR 'chronic myeloid leukemia':ti,ab,kw OR 'cml':ti,ab,kw OR 'myeloproliferative neoplasms':ti,ab,kw OR 'myeloproliferative disorder'/exp OR 'myeloproliferative disorder'/de OR 'mpn':ti,ab,kw) AND (('mutation'/exp OR 'mutation'/de) AND ('genetics'/exp OR 'genetics'/de) OR 'mutat*':ti,ab,kw OR 'genetic alterat*':ti,ab,kw OR 'somatic mutat*':ti,ab,kw OR 'variant':ti,ab,kw OR 'variants':ti,ab,kw OR 'delet*':ti,ab,kw OR 'loss of heterozygosity':ti,ab,kw OR 'loh':ti,ab,kw) AND [english]/lim
MedLine Ultimate	Filtered to English language: (XB Additional Sex Combs Like 1 OR XB asxl1 OR XB asxl1 mutat* OR XB asxl1 alterat* OR XB asxl1 delet* OR XB asxl1 loss) AND (XB MH "Leukemia, Myeloid, Chronic-Phase" OR XB Chronic Myeloid Leukemia OR XB Myeloproliferative Neoplasms OR XB MPN OR XB Myeloproliferative Disorder OR XB CML OR XB BCR-ABL1 positive MPN) AND (XB MH "Mutation" OR XB mutat* OR XB genetic alterat* OR XB somatic mutat* OR XB variant OR XB variants OR XB delet* OR XB loss of heterozygosity OR XB LOH)
PubMed	(("Additional Sex Combs Like 1"[Title/Abstract] OR "asxl 1"[Title/Abstract] OR "asxl1 mutat*"[Title/Abstract] OR "asxl1 alterat*"[Title/Abstract] OR "asxl1 delet*"[Title/Abstract] OR "asxl1 loss"[Title/Abstract]) AND ("leukemia, myelogenous, chronic, bcr abl positive"[MeSH Terms] OR "Chronic Myeloid Leukemia"[Title/Abstract] OR "CML"[Title/Abstract] OR "Myeloproliferative Neoplasms"[Title/Abstract] OR "Myeloproliferative Disorders"[MeSH Terms] OR "MPN"[Title/Abstract]) AND ("mutation/genetics"[MeSH Terms] OR "mutat*"[Title/Abstract] OR "genetic alterat*"[Title/Abstract] OR "somatic mutat*"[Title/Abstract] OR "variant"[Title/Abstract] OR "variants"[Title/Abstract] OR "delet*"[Title/Abstract] OR "loss of heterozygosity"[Title/Abstract] OR "LOH"[Title/Abstract])) AND (english[Filter])

Abbreviations: CP, chronic phase; AP, accelerated phase; BC, blast crisis; BP, blast phase; CCyR, complete cytogenetic response; EFS; event-free survival; FFS, failure-free survival; MMR, major molecular response; NGS, next-generation sequencing; OS, overall survival; PCR, polymerase chain reaction; PFS, progression-free survival; TKI, tyrosine kinase inhibitor.

Table S2. Study characteristics.

	Country	Study design	Population (age)	Total number	Disease phase(s)	ASXL1 detection method	ASXL1 mutations, n (%)	Treatment era / TKI exposure	Outcomes among AXL1 cohorts	TKI resistance (if reported)
**Shanmuganathan et al. (2025)** [17]	Australia, New Zealand	Retrospective	Adults (median 55 yrs)	515	CP	PCR/NGS	40 (8%)	Mixed	MMR at 12 months: ASXL1 55% vs other mutations 66% vs no mutations 83%, P = 0.033.<br>EFS "At 2 years (ELN 2020 definition): 61% vs 91%, P < .001".	TKI-resistant mutations at 2 years: ASXL1 mutations 35% vs other mutations 2% vs no mutations 1%, P < 0.001.
**Mohammed et al. (2023)** [18]	Iraq, Iran	Retrospective cross-sectional analytic study	Adults (median 42.6 yrs)	80	CP, AP, BP	NGS	9 (11.3%)	Mixed TKIs	MMR at 12, 18, 24: 0% for ASXL1 mutant patients.	Resistance to Imatinib: 9/9 (100%) vs 26/71 (36.62%), P=0.01.<br>Resistance to Nilotinib: 4/9 (44.44%).
**Bidikian et al. (2022)** [19]	United States	Retrospective multicenter observational cohort	Adults (median 60 yrs)	115	CP, AP, BP	NGS	21 (18.3%)	First- and later-generation TKIs	CCyR:<br>CP-ASXL1: 8/9 (89%);<br>BP-ASXL1: 1/5 (20%);<br>AP-ASXL1: 2/6 (33%).<br>Cumulative MMR:<br>CP-ASXL1, 7 (78%); median time to MMR, 17.5 months;<br>BP/AP-ASXL1, NA.<br>OS: BP-ASXL1, 7.2 months;<br>AP-ASXL1, 25.7 months.<br>5-year PFS: CP-ASXL1, 88%; BP-ASXL1, 0%; AP-ASXL1, 24%.	ASXL1 mutants: ~45–50% failed to achieve MMR.
**Schönfeld et al. (2022)** [20]	Germany	Prospective cohort (clinical trial associated)	Adults (median 52 yrs)	222	CP	NGS	20 (9.0%)	Frontline imatinib based	MMR at 12 months: ASXL1 55% vs no mutation 85%.<br>MMR at 18 months: ASXL1 60% vs no mutation 89%.<br>MMR at 24 months: ASXL1 65% vs no mutation 89%,<br>P < 0.008.	

**Hu et al. (2022)** [21]	China	Retrospective observational cohort	Adults (median 50 yrs)	22	CP	NGS	9 (40.9%)	Mixed TKIs	MMR at 12 months → no difference. MR4.0 at 36 months → significant inferior response in patients with ASXL1 mutations.	
**Romzova et al. (2021)** [22]	Czech Republic	Prospective observational cohort	Adults (median 64 yrs)	49	CP	NGS	6 (12.2%)	Mixed TKIs	The study concludes that the presence of ASXL1 at diagnosis did not significantly predict a poorer molecular response or a higher rate of treatment failure in this Czech patient cohort.	
**Ochi et al. (2021)** [23]	Japan	Multicenter cohort	Adults (median 45 yrs)	216	CP, BC	NGS	33 (15.3%)	Mixed TKIs	For patients with ASXL1 mutations, progression to blast phase was significantly shorter than for wild-type patients (hazard ratio: 4.66 (95% CI: 1.99–10.89), P < 0.001).	
**Awad et al. (2020)** [24]	Finland, Egypt	Retrospective genomic cohort	Adults (median 52 yrs)	59	CP, AP	NGS	11 (18.6%)	Mixed TKIs	ASXL1 with other mutation had poor outcomes (exact CCyR and MMR for ASXL1 not reported).	
**Wu et al. (2020)** [25]	China	Cross-sectional genomic analysis	Adults (median ~38–52 yrs)	63 (all resistant/ intolerant of TKIs)	CP/AP	NGS	15 (23.8%)	TKI-intolerant/ resistant cohort	Neither CCyR/MMR was studied. ASXL1 mutation was associated with adverse clinical features at diagnosis and may have contributed to disease progression; however, independent impact on progression-free survival was not demonstrated.	
**Branford et al. (2018)** [9]	Australia, Germany, UK, Korea	Retrospective genomic cohort	Adults (median 49 yrs)	65	CP, BC	NGS	9/65 (13.8%)	Frontline imatinib	MMR3 in 2/9 ASXL1 patients. 6/9 progressed to BC	7/9 (78%) had poor outcome/TKI failure.
**Kim et al. (2017)** [26]	South Korea	Retrospective cohort	Adults (median 55 yrs)	100	CP, AP, BP	NGS	9 (9.0%)	Imatinib based	At 12 months, treatment failure or failure to achieve or maintain CCyR occurred in ASXL1: 5/9 (55.6%), vs other mutations: 14/28 (50%), vs no mutation: 7/63 (11.1%); P = 0.015<br>At 24 months, achieved MMR in ASXL1: 4 (44.4%), vs other mutations: 14 (50.0%), vs no mutation: 56 (88.9%); P = 0.092	

Figures

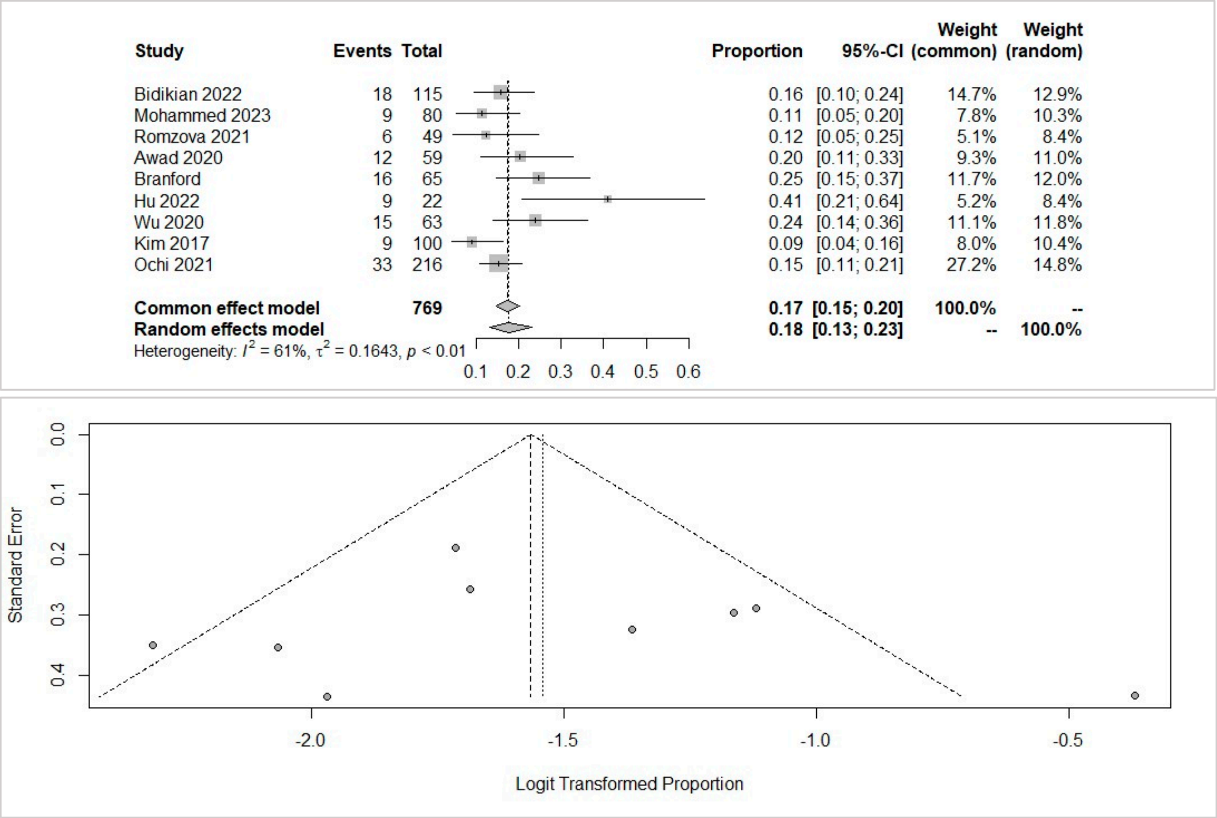

Figure S1. Forest and funnel plots for the prevalence of ASXL1 mutations. Sensitivity analysis by removing studies by Schönfeld 2023 and Shanmuganathan 2025 (i.e., patients with CP-CML).

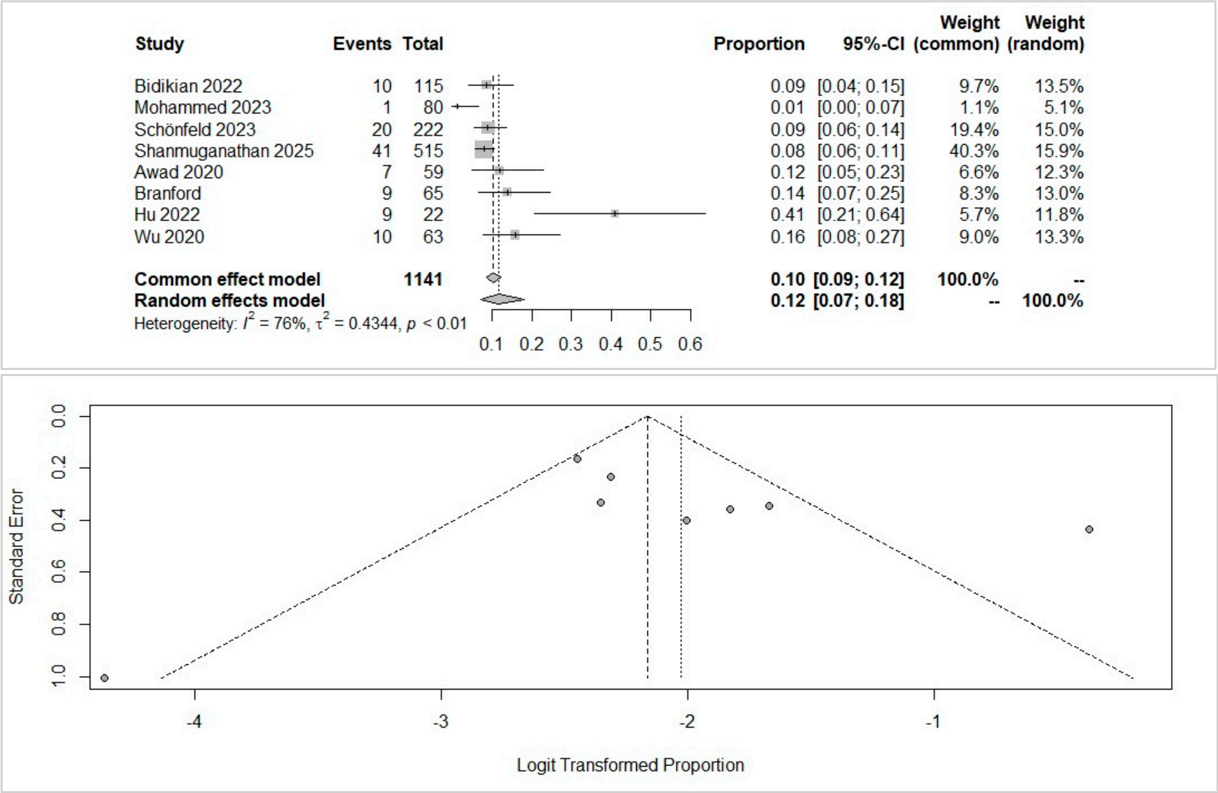

Figure S2. Forest and funnel plots for the prevalence of ASXL1 mutations in CP-CML.

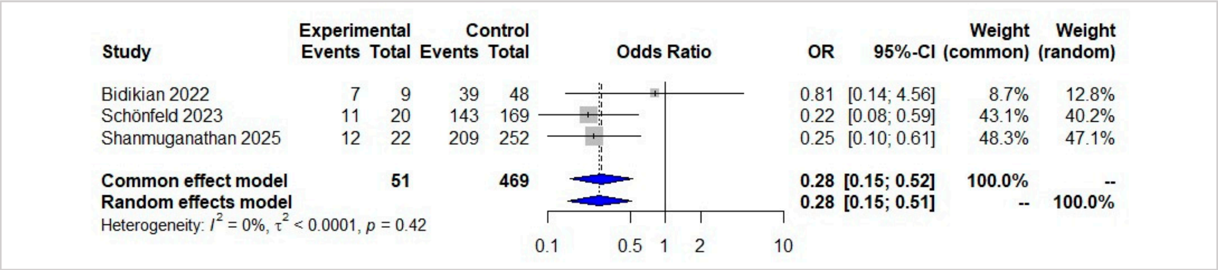

Figure S3. MMR—Sensitivity analysis using studies by Bidikan, Schonfeldl, and Shanmuganathan et al. (i.e., patients with CP-CML only).

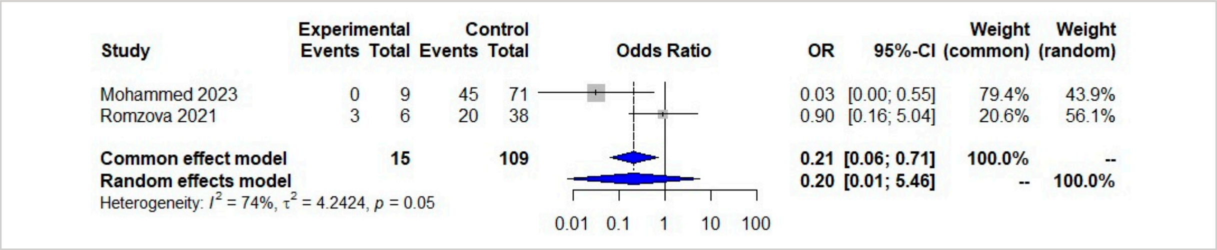

Figure S4. MMR—Sensitivity analysis by removing studies by Bidikan, Schonfeld, and Shanmuganathan et al. (i.e., patients with CP-CML).

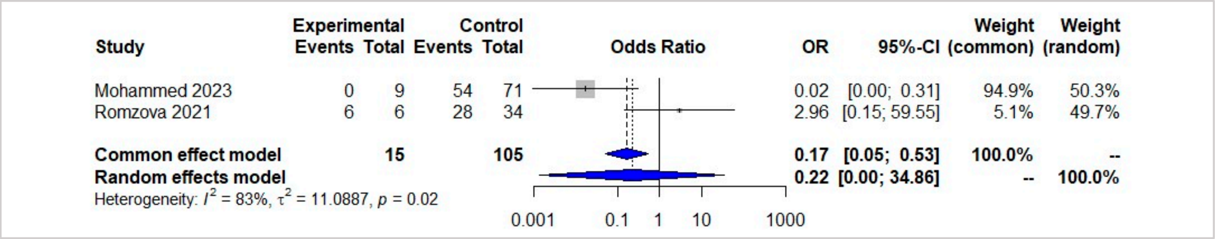

Figure S5. CcyR—Sensitivity analysis by removing study by Bidikan et al. (i.e., patients with CP-CML).
